# Explainable AI as evidence of fair decisions

**DOI:** 10.3389/fpsyg.2023.1069426

**Published:** 2023-02-14

**Authors:** Derek Leben

**Affiliations:** Tepper School of Business, Carnegie Mellon University, Pittsburgh, PA, United States

**Keywords:** xAI, explainability, fairness, discrimination, counterfactual explanations

## Abstract

This paper will propose that explanations are valuable to those impacted by a model's decisions (model patients) to the extent that they provide evidence that a past adverse decision was unfair. Under this proposal, we should favor models and explainability methods which generate counterfactuals of two types. The first type of counterfactual is *positive* evidence of fairness: a set of states under the control of the patient which (if changed) would have led to a beneficial decision. The second type of counterfactual is *negative* evidence of fairness: a set of irrelevant group or behavioral attributes which (if changed) would *not* have led to a beneficial decision. Each of these counterfactual statements is related to fairness, under the Liberal Egalitarian idea that treating one person differently than another is justified only on the basis of features which were plausibly under each person's control. Other aspects of an explanation, such as feature importance and actionable recourse, are *not* essential under this view, and need not be a goal of explainable AI.

## 1. Introduction

In a 2016 story titled: *Rejected for Credit? Newfangled scores may be to blame*, the LA Times described the case of a Huntington Beach resident named Joseph who had an excellent FICO credit score of 820, yet was still rejected for a Bank of America credit card. In the U.S., the Equal Credit Opportunity Act (ECOA) requires companies to disclose the “specific reasons” why applicants were rejected for credit, and in the letter which Joseph received from Bank of America, they mentioned that the decision was partly based on a score called his Credit Optics Score, which was produced by another company called SageStream. The article describes that Joseph contacted SageStream to ask about how this score was calculated, but could not receive any satisfactory information about the CreditOptics model. Indeed, in an article in Business Wire, a representative of SageStream described that the model they use is based on a convolutional neural network architecture, and trained on a large data set from the ID Analytics Network, which includes “data not typically analyzed in traditional credit scores, including transaction data from wireless, cable and utility accounts; online marketplace, payday and subprime lending; and other credit-relevant alternative data sources”. Given all this, it might be that the reason why SageStream did not inform Joseph about how the CreditOptics model arrived at its decision is that the model and data they were using are so complex that it would be difficult for even the engineers who designed it to give a good explanation for why it rejected his application.

The Problem of Explainable AI (xAI) is that complex models built with machine learning methods provide very powerful tools for predictive accuracy, but they are often “black boxes”, meaning that their internal operations are not easily interpretable. This problem has created a growing demand in both the ethics and regulation of AI for explainability, especially in high-stakes domains (Jobin et al., 2019).

In response to the legal and ethical demand for explainability, there has been a surge in research which has produced a large set of xAI methods in a short period of time. These xAI methods differ in the type of explanations which they are capable of generating about the decisions of a model, and to evaluate an xAI method it now becomes necessary to ask: what type of explanations are important for an xAI method to produce?

This paper will propose a normative and psychological claim about the value of explanations for those who are impacted by the decisions by an AI model (model patients). Call this the *Evidence of Fairness* view:

**Evidence of Fairness:**

*Explanations are valuable to model patients because they are evidence that a past decision was fair*.

According to this claim, people can and should care about explanations to the extent that those explanations provide compelling evidence that a past decision (almost always an adverse decision) was fair. Furthermore, it turns out that certain types of explanations about *counterfactual states* (CS) are the type of explanations which best accomplish this. CS-explanations can show that the model which made a decision *would have* produced a beneficial decision under alternative conditions which are under the control of the model patient (which we'll call “positive evidence of fairness”). CS-explanations can also show that the model *would not have* produced a beneficial decision when we alter irrelevant behavioral or group attributes (which we'll call “negative evidence of fairness”).

The paper is structured as follows. Section 2 will describe the goal of explainable AI as justifying the decision of a model, and identify three types of explanations which xAI methods generate: feature importance (FI), counterfactual state (CS), and actionable recourse (AR). Examples will mostly draw on the domain of credit scoring, since this is the area which is most heavily regulated with respect to explanations (in the U.S., applicants who are rejected for credit applications are owed an explanation, while applicants are not necessarily owed an explanation in other domains like hiring, medicine, marketing, etc.). Section 3 will present reasons to believe that people care deeply about the fairness of a past adverse decision about them, and how both psychological and normative theories evaluate fairness in terms of counterfactuals. Section 4 will show how CS explanations can provide two types of evidence of fairness. The positive variety is evidence that controllable counterfactuals *would* have led to a better decision, while negative variety is evidence that irrelevant counterfactuals *would not* have led to a better decision. Section 5 will argue that the two other types of explanations (FI and AR) are not essential for satisfying explainability under the Evidence of Fairness view. The concluding section will consider practical implications for both technical metrics and regulations in the domain of xAI.

## 2. Explainability

The research on xAI has moved so quickly that there are now not only meta-reviews which propose taxonomies for xAI methods (Arrieta et al., 2020; Angelov et al., 2021; Belle and Papantonis, 2021), but meta-meta-reviews which propose taxonomies of these taxonomies (Vilone and Longo, 2021; Speith, 2022). These taxonomies distinguish xAI methods by categories like what sorts of AI models can be explained by an xAI method (i.e., are they *model-agnostic* or *model-specific*), what parts of a model can be explained by an xAI method (i.e., are they *global* or *local*), and at what stage the xAI method interacts with the AI model (*ante-hoc* or *post-hoc*). For our purposes, what we care about are the *outputs* of an xAI method, and whether these outputs provide the right kind of explanations for the decisions of a model. As such, when we talk about an “explanation” we are referring to the output of an xAI method.

The output of an xAI method can be represented in many ways, such as a graph or table of features with associated values, a visual diagram like a heat map, or even a text string (e.g., “your loan was rejected because your income is too low”). But for our philosophical and psychological purposes, it is most useful to think about how the designer of the xAI method would “read out” the output of that method to a user. Thus, we are focusing entirely on the “intended use” of the xAI method, and bypassing the important challenge of how designers should communicate these intentions to users. For example, looking at a saliency map, a designer would read this as: “here are the features which had the largest marginal contribution to the decision of the model”. When we say that an xAI method “generates” an explanation, we will mean that this is how an xAI designer could read off the outputs of an xAI method to a user.

We can identify three different types of explanations that an xAI method might generate:

**Feature Importance (FI):**

The fact that your income was &40k was the most significant factor in your rejection, being roughly 30% of the negative contribution from the original neutral starting assumptions.

**Counterfactual State (CS):**

If your salary had been higher than &50k (all else being equal), then you would have been approved for the loan.

**Actionable Recourse (AR):**

The best method for you to improve your future credit score, according to our model, is by paying off your highest-debt credit card and increasing your savings beyond &5k.

The distinction between FI and CS types is emphasized by Kusner et al. (2017), Wachter et al. (2018), and Miller (2019), who note that xAI methods which generate FI explanations do not always generate CS explanations, and vice-versa. Sometimes the distinction between FI and the other types is called a difference between explanations by “features” and explanation by “examples” (McDermid et al., 2021), but the latter term is not very informative. The distinction between CS and AR explanations has been emphasized by Ustun et al. (2019) and Karimi et al. (2021), who note that an xAI method which generates CS explanations does not always generate AR explanations. Haynes et al. (2009) call FI outputs *mechanistic explanations* which answer the question “how does it work?” while AR outputs are *operational explanations* which answer the question “how do I use it?” Of course, the CS layer in between (which they do not identify) is something like: “how would it work differently?” These distinctions also have a historical ancestry in the philosophy of science debate about what constitutes a good causal explanation.^1^

Some models and xAI methods can generate all three types of explanations. For example, the classic FICO model, developed in 1956 by the engineer Bill Fair and mathematician Earl Isaac, is a simple linear model, where data from an applicant is assigned a numerical code, multiplied by weights, and added together (along with a bias term) to produce a score somewhere between 350 and 850. The details of the model are proprietary, but the company has revealed that there are five data types (or dimensions), which have the corresponding weights:

- 35% Payment History
- 30% Amounts Owed
- 15% Length of Credit History
- 10% New Credit
- 10% Credit Mix

It is easy to generate explanations of every type for why an applicant was rejected by this model. The list above is a straightforward FI-explanation. But even further, because the model is linear, a change in the features has a proportional effect on a change in the score, so we can easily generate CS-explanations like: “a decrease in the amounts owed by *x* will have a 0.30(*x*) positive influence on your score”. The applicant can use her knowledge of which features are easiest to change to generate AR-explanations based on maximizing the changes which are both easily controlled and produce the highest impact.^2^ Importantly, credit companies in the U.S. have historically only provided the first type of explanation, and allowed consumers to calculate the other two types for themselves. Thus, when they are compliant with a law to “provide explanations”, one reading is to provide the *materials* which can be used by a reasonable person to generate explanations (this “reasonable person” standard is also important for obligations about products and advertising).

When an explanation can be generated from a model without the use of an xAI method, we can say that the model is *inherently explainable*. For example, linear regression and decision-tree models are inherently explainable. The FICO model is inherently explainable, because it is possible to derive each type of explanation from it without the use of an xAI method. On the other hand, complex models like deep neural networks, support vector machines, and random forests are not inherently explainable. The CreditOptics model is a convolutional neural network trained on a large set of non-independent features, so it is not inherently explainable in the way that the FICO model is. Thus, we must apply an xAI method to try to generate at least one type of explanation from it. Yet, given that some xAI methods are capable of generating only some of these explanation types, the question of “which xAI method should we use” becomes the question: which explanation types are the ones that should be preferred over others?

An influential account of explanations from the philosophy of science is known as the *Pragmatic Theory of Explanation* (VanFraasen, 1980; Achinstein, 1983), where the explanatory value of some claim for another set of claims, facts, or observations depends on the interests of people evaluating that claim. The Pragmatic Theory of Explanation has been extended into xAI by authors like Langer et al. (2021) and Lu et al. (2021), and we will follow suit. As Adadi and Berrada (2018) note, different stakeholders may have different reasons to care about explainability, where academics may be more interested in xAI methods and their outputs for discovery and teaching, while engineers are more interested in xAI methods and their outputs for understanding and controlling the model. In this paper, we are centrally concerned with the value of people who are impacted by an AI model's decisions, to whom we refer as “model patients” (rather than the term “data subject”, which is often ambiguously used to refer to those whose data are used to generate a model and those who are impacted by a model's decisions, which are not always the same groups). The proposal here is that the primary interest of model patients (both empirically and normatively) is to determine if the decisions of a model were justified, rather than to understand how the model works. In an experiment presenting people with credit decisions from more and less inherently explainable models (shallow decision trees vs. neural networks), Lu et al. (2021) found that people were *less* satisfied with an explanation in the form of an easily understandable decision-tree compared with explanations generated by xAI methods which do not reveal the operations of the model itself.

Thus far, we have been keeping one foot in the empirical and another in the normative, by saying that people “can and should” care about explanations because they are evidence of fairness. This is deliberately avoiding tricky issues with the divide between the two (the “is/ought gap”). For most of our discussion the two claims will be in harmony: the Evidence of Fairness view is both a psychological account of why people care about explanations, and also a normative account of why people have a right to explanations. This is partly why it is being called a “view”, to emphasize the dual role as both psychological description and normative prescription. The argument for this view as a psychological theory is speculative, it is offered as a plausible account of why people care about explainability for automated systems like CreditOptics. However, the argument will be bolstered by a normative account of why people *should* care about explainability in automated systems, regardless of why they actually do.

Generally, the approach adopted here for addressing the is/ought gap is Greene's (2013) meta-ethical framework where normative principles emerge naturally and historically as rationalizations of intuitive moral judgments, but can then move beyond that into solutions for cooperation problems in large-scale societies. Thus, there is an important etymological connection between intuitive judgments of fairness and normative theories of fairness, but the latter can be evaluated by new criteria beyond their mere acceptability. This is also similar to Rawls' (1971) idea of “reflective equilibrium”, where a theory of fairness must both solve cooperation problems and also bear some fidelity to our intuitive moral judgments. The main difference between Greene and Rawls is the former's willingness to disregard the considerations of intuitive moral judgments when need be. However, assuming that the Evidence of Fairness view correctly describes both people's actual concerns about explainability as well as their best reasons for caring about explainability, there is no need to resolve any conflict between the two. Indeed, the normative account can further “flesh out” the skeletal intuitions which people often have about explainability.

## 3. Fairness

A decision being perceived as fair or unfair has an important impact on human behavior. In the famous Ultimatum Game, participants are willing to make financial sacrifices (sometimes very large ones) when they perceive a decision about them to be unfair (Nowak et al., 2000). This is not to suggest that most people have an explicit and formal theory of fairness which they use to evaluate outcomes. Instead, it is likely that most people have an intuition that, as Aristotle described, fairness means treating “equals equally and unequals unequally”, without specifying the details of when recipients should be treated equally and why. It is this vague intuition which normative theories of fairness seek to develop and articulate. This section will provide some reasons for believing that fairness intuitions play a large role in people's satisfaction with outcomes (including those produced by automated systems), and the normative theory that we will be using to show how these desires for fairness can be formally implemented into automated decision-making.

Perceptions of fairness have a well-established effect on attitudes about a company's decisions in the minds of employees (Cropanzano and Greenberg, 1997; Loi et al., 2009), consumers (Xia et al., 2004), and job applicants (Gilliland et al., 2001). Folger and Cropanzano attempted to provide a theoretical account of the psychological mechanisms driving these effects in what they called *Fairness Theory* (Folger and Cropanzano, 1998; Nicklin et al., 2011; Nicklin, 2013). According to Fairness Theory, judgments of fairness are driven by counterfactual reasoning about three domains, which they term “would”, “could”, and “should”. The first counterfactual involves an evaluation of harm through consideration of alternate outcomes, where a negative change has occurred in the actual world which would not have otherwise happened. The second counterfactual involves an evaluation of responsibility through consideration of alternatives consistent with the agent's abilities, under the familiar ethical concept that an agent is responsible to the extent that she could have done otherwise. Finally, the third counterfactual involves an evaluation of norm violation through consideration of alternatives where some fairness metric or principle is followed. For our purposes, we will assume that some obvious adverse impact has occurred, such as the denial of a loan, and the question about fairness will center around evaluating counterfactuals about responsibility and norm violation.

When it comes to responsibility attribution, the two key features that people tend to care about are *intentionality* and *preventability* (Malle et al., 2014). When an action is viewed as unintentional, the question of responsibility then becomes entirely an issue of preventability (Monroe and Malle, 2017). For example, in Monroe and Malle's experimental paradigm, they contrast the following three cases:

- Ted hit a man intentionally with his car for no reason
- Ted hit a man intentionally with his car for good reason
- Ted hit a man with his car unintentionally, because he didn't check his blind spot
- Ted hit a man with his car unintentionally, because his brakes failed

In the latter two cases, the question turns from intentionality to whether (and to what extent) the outcome was within Ted's power to prevent. Judgments about whether an action was “within one's power” can be evaluated with a counterfactual where we imagine alternative worlds where the agent attempted to perform some action. In alternative worlds where everything from our world is held constant except Ted checking his blind spot, the man would not have been injured by his car, so most people attribute a high degree of responsibility to Ted, even though he did not intend the harm. Even in the case of the car brakes failing, one might still blame Ted for not checking his brakes, although this is more difficult for him. One interpretation of these effects is that responsibility judgments are influenced by the patient's practical ability to change an outcome. These results are also found in experimental tests of judgments about fair distribution from differences in practical ability (Alexander et al., 2010; Micheli and Gagnon, 2020).

In addition, people tend to focus on a small set of agents when considering counterfactuals about responsibility. In the example of Ted hitting a man with his car, this bad outcome could have also been prevented by other agents acting in different ways which were within their power. If Ted's wife had asked him to pick up bagels on the way home from work, he wouldn't have hit the man, but it's strange to attribute responsibility to Ted's wife. On the other hand, if Ted had his brakes checked the day before, and the mechanic had not correctly done her job, then this might cause a shift in blame from Ted to the mechanic, which is an effect called “blame blocking” (Cushman, 2008). This is an important effect, because when there is a perceived harm which is also a violation of norms, people are eager to attribute responsibility to some party, and the question becomes less one of “did Agent *X* have the power to prevent this?” and more a question of “*which* agent had it within their power to prevent this?”

The normative component of fairness judgments has to do with whether the actions which brought about an adverse impact were justified. The counterfactual evaluation of justification here is: which changes that bring about an adverse impact (and are intentional/preventable) are permissible, and which are impermissible? As Folger and Cropanzano (1998) note, there are a variety of norms for evaluating fair outcomes, including equality, desert, and benefit, but these norms are often applied selectively and inconsistently (Jones, 1991). Thus, to expand our analysis to a coherent framework which can clearly define which sorts of counterfactual changes are compliant with fairness norms, we should appeal to a normative theory [much like Greene's (2013) suggests].

The normative theory which we will employ here is Liberal Egalitarianism, as developed by 20th century philosophers like Rawls' (1971), Dworkin (1981), Cohen (1989), and Roemer (1998). This theory is an attempt to resolve the tension between the values of liberty and equality by specifying in which domains people should be treated equally, and what counts as the qualifying conditions for unequal treatment. According to Rawls' version of this theory, there must be equal distributions of rights and what he calls “primary goods”, which are the conditions for pursuing the goals that any person might potentially have. However, the distribution of “secondary goods” may be on the basis of both merit and luck.

This has important implications for debates about goods like healthcare and insurance, where Liberal Egalitarians typically argue that a just society should provide all citizens with emergency medical services, regardless of features like desert. If a drunk driver collides with another car and both people are injured, both patients should be provided with medical treatment. Indeed, if the drunk driver's injuries are severe and the passenger of the other vehicle's injuries are minor, the drunk driver should be treated first, regardless of merit (this has become especially relevant in recent debates about whether vaccinated and non-vaccinated patients should both receive equal priority in the distribution of scarce resources like ventilators). However, Liberal Egalitarians may also permit private insurance companies to charge higher rates for smokers than non-smokers on the basis of merit-based considerations. In Pew Surveys, a majority of Americans favor charging higher insurance rates for smokers over non-smokers, while a minority of Americans favor charging higher rates for overweight vs. non-overweight people. Both of these traits are relevant to the likelihood of healthcare costs, but one trait is often perceived as a more permissible qualifying characteristic.

One of the most popular Liberal Egalitarian accounts of what counts as a qualifying characteristic is that qualifying traits are those over which people have a greater degree of voluntary control, as opposed to what we might broadly call “luck” (Cohen, 1989). For example, people may arguably have a greater degree of control over the fact that they are a smoker than the fact that they are overweight, and it is therefore permissible to charge them more for insurance. One fascinating result of this account is that it connects responsibility and norm evaluations together; the fairness of an outcome is in some sense reduced to a person's responsibility for that outcome (only for domains where egalitarian principles are not required). This account is very good at explaining why it is wrong to discriminate based on protected attributes like race and gender (these features are obviously difficult to change), and there is some research suggesting that lack of control plays a role in people's judgments about fair distributions (Tinghog et al., 2017). However, we need not consider voluntary control as the only possible account of qualifying features; one could also use a broader task-relevance account which evaluates features based on the purpose of an activity (Halldenius, 2017). Both the control and relevance accounts would rule out the use of protected features like race and gender, but the latter would also potentially rule out features like how many children a person has or how long they charge their cell phone at night (an increasingly serious risk when it comes to AI models trained on big data).

By adopting Liberal Egalitarianism as our theory of fairness (both empirically and normatively), we are excluding some alternatives that should be noted. Most obviously, we are ignoring more extensive versions of both Liberalism [e.g., all distributions should be made on the basis of merit and luck considerations (Nozick, 1981)] and Egalitarianism [e.g., all distributions should be made on the basis of equality considerations such as ability and need (Anderson, 1999)]. In the above example of health insurance coverage and costs, we are thus ignoring the “extreme” views that people should have to pay higher costs because of medical conditions which are beyond their control, and also that people should be provided with equal insurance costs and coverage regardless of their medical conditions. In our other example of decisions about credit and lending, we are obviously ignoring the view that all people should receive equal credit scores, but also (and far more importantly) the view that credit scores should be based on features which might be statistically related to the ability to repay a loan but are beyond the control of an applicant, like the highest educational status of one's parents, or the credit scores of one's family and social network. These features are increasingly being used as “alternative credit data” by companies like SageStream, and Liberal Egalitarianism takes a clear position that these features are unfair to use in credit decisions.

Another approach to fairness which is being excluded here is the Utilitarian approach, where fair distributions are those which produce more overall benefit (Trautmann, 2010; Hooker, 2017). For Utilitarians, whether a set of features should be used in making decisions about insurance or credit depends on the positive and negative impacts which use of those features would have on companies, customers, and the general public. Fairness is thus, like all things, reducible to utility. The use of protected attributes like gender and race tends to create negative social impacts, but the use of features like highest educational status of parents might not, so it is possible that Utilitarians would allow credit models that make use of the latter. Utilitarian calculations are, of course, extremely complex; the use of parent's highest educational status could indeed create more long-term suffering by continuing to keep children of poor families in poverty, and these are important factors for the Utilitarian to consider. The point here is that Liberal Egalitarians do not engage in these sorts of welfare calculations, and instead they can judge that features like late payments are always fair and features like parent's education are always unfair, regardless of outcomes on individual and social welfare.

Finally, the Liberal Egalitarian is committed to an *individualistic* and *procedural* sense of fairness rather than one that focuses on groups and historical considerations such as repairing past injustices. When all large subsets of a population contain equal distributions of relevant qualifying traits, there is no conflict between individual and group fairness. However, when one group has more qualifying traits than another (most likely because of historical injustices), then there can be a conflict between these considerations. For example, according to the Brookings Institution, in the U.S. in 2019 the median amount of wealth (assets to debt) for Black families was about &24K, compared with &188K for White families. If we are only considering “relevant” qualifying attributes related to wealth, then White applicants will likely be rewarded with credit at much higher rates than Black families. Attempting to enforce a simple group fairness metric (like demographic parity) between Black and White applicants for credit scores or lending rates will inevitably lead to violations of individual fairness. This is especially relevant for automated systems, since many of the methods for evaluating fairness in AI involve group parity metrics (Barocas et al., 2019). This conflict between correcting historical injustice between groups and preserving procedural fairness to individuals is of course at the heart of Affirmative Action debates (which often apply in admissions and hiring decisions, although historical injustice arguments could just as easily apply to credit and lending). Liberal Egalitarians have often disagreed about how to resolve this conflict. Dworkin (1981) and Nagel (2003) both defend Justice Powell's famous 1978 opinion that group membership can be a qualifying factor when other qualifying factors are satisfied (in the U.K. these are known as “tie breakers”). Thus, individual fairness still takes priority over group fairness. We'll return to this problem in the concluding section, where we consider how the xAI methods for justifying individual fairness compare with other popular methods for justifying group fairness.

## 4. CS explanations as evidence of fairness

According to the Evidence of Fairness view, the value of explainability for AI models is driven by the value of procedural justice in important decisions. Demands for procedural justice in human-made decisions also extend to demands for procedural justice in automated decisions (Otting and Maier, 2018), but there are important differences in people's expectations about the latter (Starke et al., 2022). Namely, many people view AI decisions as more likely to miss important qualifying features (Newman et al., 2020), and less likely to make use of irrelevant protected features (Claudy et al., 2022). As these authors observe, this may be due to the widespread assumptions about automated systems that they are incapable of traditional bias against protected groups, and that many qualifying features are incapable of being quantified and evaluated computationally. Yet both these assumptions are false. This is partly why it is necessary to move beyond laypeople's intuitions about automated systems and into normative reasoning guided by a theory of fairness, while still attempting to preserve some degree of “reflective equilibrium” with people's intuitive attitudes.

Within a Liberal Egalitarian framework, there are two specific types of CS-explanation which are of pragmatic importance in evaluating whether a decision was fair: (1) demonstrating that there are “controllable” changes which could have been made to produce a beneficial outcome, and (2) demonstrating that “irrelevant” behavioral or group attributes would not have produced a beneficial outcome. The next two subsections will focus on each of these types of CS-explanation, framing them as the sort of evidence that will show that a decision was fair.

### 4.1. Positive evidence: Sensitivity to controllable counterfactuals

There are a massive number of counterfactual changes to a model patient which would lead to a better outcome for her, yet people tend to focus on a very small subset of these. Byrne (2019) emphasizes that explanations for adverse impacts which people find satisfying are mostly concerned with counterfactuals over which patients had some kind of power, and which are most easily accomplished. Let's call this “positive evidence” of fair decisions.

In experiments by Girotto et al. (2019), they presented people with stories like the following and asked them to complete the counterfactual statement at the end:

Anna, an undergraduate at your university, was asked to participate in a game. A research assistant told her, “In order to win two chocolates, you have to mentally multiply either two one-digit numbers or two two-digit numbers, in 30 seconds. If you fail, you do not receive the chocolates”. The two multiplication problems are contained in two sealed envelopes. Let us call them envelope A and envelope B. Of course, we do not know which envelope contains the one-digit multiplication problem and which one contains the two digit multiplication problem. Anna accepted the offer to participate. She chose envelope A, and the research assistant opened it. Unfortunately, it contained the two-digit multiplication problem. She failed. Things would have been better for Anna, if …

Most people complete this counterfactual by saying that things would have been better for Anna if she had chosen a different envelope. However, there are other counterfactual factors which are just as relevant to her bad outcome, such as her inability to calculate a large number quickly, or the fact that the research assistant did not let her use a calculator. Yet these are not factors which are under Anna's control, so they are less relevant to an explanation. It seems here that the “best” explanation for Anna's bad outcome is her choice of envelope, even though many other factors also explained the result.

One reason why more controllable counterfactual states might increase the perceived fairness of a decision is that an action being *within one's power* is a way of evaluating the preventability of an outcome, which we've seen is a key factor in fairness judgments. A related reason why more controllable counterfactual states may increase the perceived fairness of a decision is that this demonstrates that a bad outcome was less the result of bad luck, and thus less justified for equal treatment. There is a large literature on the psychology of reasoning about control and agency (Gallagher, 2000), and we might simply leave this as a variable to be filled in accordingly: “the counterfactuals which provide better positive evidence of fairness are whatever changes people view as more under the patient's control than others”.

It is important to note that the states which are under a person's control relate to both physical possibility (what she “has power over”) and also practical possibility (what she “can easily accomplish”). While it might seem like these are discrete categories, or even identical categories, Kratzer (1977) has argued that these are both scalar and distinct. Specifically, they refer to the set of possible worlds which are more coherent with a set of assumptions: physical possibilities are the worlds more coherent with the laws of nature, and practical possibilities are those more coherent with a person's goals and interests. Kratzer is looking to explain the semantics of words like “can” and “able”, where people often say “I am not able to make the meeting on Wednesday because of a dentist appointment,” and mean something like “Wednesday's meeting is not coherent with my other goals.” Indeed, it is common to use terms like “better able to make a meeting on Thursday” to suggest that this is a state which is more consistent with one's other interests. Thus, one might say it is physically possible for a person to both pay off &2K in debt or change careers, but given that person's goals and interests, one of these changes is more practically possible than the other (within the literature on counterfactuals, these terms are usually defined in terms of “nearby” or “distant” possible worlds).

If controllability is indeed a scalar concept, then we can measure the magnitude of good positive CS explanations in terms of how practically controllable some counterfactual state was for the model patient. The following three CS explanations can be ranked in order of better and worse positive evidence of a fair decision:

*You would have been approved for the loan if you had*…

1. Paid &2k off your existing debts
2. Paid &5k off your existing debts
3. Paid &5k off your existing debts and changed careers

The reason why we can easily rank these in order (1,2,3) is that they become increasingly less easily accomplished by the model patient, not in terms of greater effort but in terms of greater sacrifice with other goals and interests. These might all be physically possible and within a patient's power, but more or less practically possible for that person.

There is some initial support for the importance of patient control in judgments about what constitutes a satisfactory explanation for automated decisions. In a set of experiments examining the causes of fairness judgments about automated decisions, Grgić-Hlača et al. (2018a,b) found that whether features are voluntary and relevant are both important factors. However, they also found a large degree of variance in these judgments, and some participants may conflate explainability with general considerations about reliability and accuracy of a model, which are distinct values. Thus, more work needs to be done to support the specific connection between judgments of control and satisfactory explanations for automated systems.

### 4.2. Negative evidence: Insensitivity to irrelevant counterfactuals

In U.S. anti-discrimination law, there is a standard which is often used called the “similarly situated persons” test, where an employer has discriminated against a person, *p*_1_, if there is another person, *p*_2_ who is similar in a high degree to *p*_1_ except for some protected attribute, and *p*_2_ has received better treatment than *p*_1_. However, it has always been difficult to establish what “similarly situated” means here, and as recently as 2019, the U.S. 11th circuit court of appeals acknowledged the vagueness of the standard:

Under that framework [the similarly-situated persons standard], the plaintiff bears the initial burden of establishing a prima facie case of discrimination by proving, among other things, that she was treated differently from another “similarly situated” individual—in court-speak, a “comparator.” Texas Dep't of Cmty. Affairs v. Burdine, 450 U.S. 248, 258–59 (1981) (citing McDonnell Douglas, 411 U.S. at 804). The obvious question: Just how “similarly situated” must a plaintiff and her comparator(s) be?

Despite the vagueness of the similarity metric, the ethical motivation behind the law is clear: protected attributes should not make a difference in how a person is treated. Here, “make a difference” is a counterfactual statement about whether a person *would have been* treated differently if a protected attribute were changed, and all other features were held constant. Indeed, the ideally similarly situated agent is clearly the same exact person, but with very slight changes to her past.

Within the context of xAI and giving evidence of fair decisions, we can think of this standard as a claim about which minimal counterfactual changes would *not* be present in states where a model approved the loan. Let's call these “negative evidence” about a fair decision. This contrasts with the discussion from the previous section of features which are in a person's control, and which are the minimal changes which *would* be present in states where a model approved the loan, which we called “positive evidence” about a fair decision. In both cases, we are interested in minimal counterfactual changes in the state of a model patient (or “nearby possible worlds”) which would be present in worlds where she is approved. We are also using the same pragmatic restriction to narrow down all the possible changes to those that are of particular interest to evaluating fairness. However, while the pragmatic restriction in positive evidence is about the changes which were relevant and/or under a patient's control, the pragmatic restriction in negative evidence is about the changes which are “protected” or “irrelevant” to the task.

In their paper, *Counterfactual Fairness*, Kusner et al. (2017) present counterfactual xAI within the framework of a similarly-situated person test for discrimination (although they do not actually cite or name this legal standard). Their claim is that, for some protected attribute, A, and prediction Y, a model is “counterfactually fair” just in case “changing A while holding things which are not causally dependent on A constant will not change the distribution of Y” (see also Wang et al., 2019). For example, the model would be fair with respect to gender if the decisions are the model are the same when we keep all the features of an applicant fixed, and merely change the gender of the applicants.

Like positive evidence of fair decisions, there can be stronger and weaker negative evidence depending on how many features we change and how much this impacts the distribution of outcomes across groups. For example, if changing the gender of an applicant from Female to Male changes the likelihood of approval by a slight amount, this is clearly better than changing the likelihood of approval by a large amount, but still not as good as no change at all (which is ideal). In addition, because there are an indefinite number of counterfactual changes which we could measure, we are pragmatically limiting these to the features which are most obviously protected or irrelevant, and features which have been the historical cause of discrimination (e.g., gender, race, age, disability, etc.). This requires data controllers who are generating CS explanations about their model to demonstrate non-discrimination to provide a priori normative reasons why they are testing the counterfactual impacts of a certain set of features.

A number of authors have objected to the use of CS explanations as evidence of non-discrimination, on the grounds that features like “race” are not simple in a way that constitutes a minimal change to a person (Kohler-Hausmann, 2019; Kasierzadeh and Smart, 2021), or that “race” is emergent from a set of other causal features which cannot be minimally changed (Marcellesi, 2013). These are important objections, and the framework of xAI as *evidence* can respond to them by agreeing that these CS explanations are not actually modulating factors like “race,” but instead, they are modulating the features which are most closely and easily predictive of race, and therefore that the CS explanation serves as *evidence* that the complex set of features which are often labeled as “race” were not likely playing a significant causal feature in the decision of a model.

## 5. Rejecting FI and AR explanations

According to the Evidence of Fariness view, the positive and negative counterfactuals described in the previous section provide both necessary and sufficient conditions for a good explanation of an adverse decision by an automated system. This section will argue that FI and AR explanations are not essential for satisfying these goals.

### 5.1. Against FI explanations

**Figure 1:**
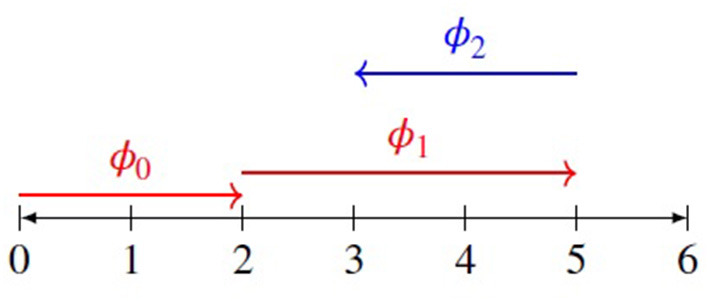
A simplified version of the visualization of SHAP from Lundberg and Lee (2017). Each feature can be assigned a value, *ϕ*_*n*_, an additive sequence toward the prediction of a model. Here, we assume that the model starts with an expectation value of 2 (caused by *ϕ*_0_), then “moves” to a score of 5 (caused by *ϕ*_1_) and finally “lands on” a score of 3 (caused by *ϕ*_2_). These shifts are not actually present in the model, but in SHAP's explanation of the model.

As examples of FI explanations, let's consider the outputs of two popular xAI methods which were both proposed around the same time: LIME (Ribeiro et al., 2016) and SHAP (Lundberg and Lee, 2017). These have been quickly adopted by many companies and organizations as a successful way to get the power of complex models like CreditOptics with the explainability of simple models like FICO. Both methods attempt to represent the features of a complex model as a set of additive weights, but it is not necessary to go into the technical details. Instead, we are concerned with how the outputs of these methods can be represented and presented to model patients. For example, Lundberg and Lee (2017) provide a helpful visual illustration of the FI-explanations generated by SHAP, which has been greatly simplified into the diagram in Figure 1.

In this simplification, we see the output of the SHAP procedure depicted as an additive sequence, *ϕ*_*n*_, of translating a decision along a credit score from 0 to 6, from the initial starting assumption about any application, *ϕ*_0_ (starting at a score of 2), to the final decision of the model, where each feature has a positive or negative magnitude which “pushes” the score to its final location along the line. For example, feature *x*_1_ exerts a “push” of magnitude *ϕ*_1_ in the positive direction, while features (*x*_1_, *x*_2_) exert a “pull” of magnitude *ϕ*_2_ in the negative direction.

It's easy to see how the FI-explanations generated by these methods look like the ones which are provided to consumers along with the FICO score. There is a list of features, along with their weighted significance. However, the important difference is that this additive list does not generate counterfactuals in the same way. For example, if we look at the SHAP visualization, it's clear that *ϕ*_2_ is causing most of the negative impact on our score. Yet there's no way to know how much we would need to change the features (*x*_1_, *x*_2_) in order to improve it. A shift like *ϕ*_2_ is not just a function of *x*_2_, but of both *x*_1_ and *x*_2_, and there's no way to know from SHAP how much a change in one of these features will change the positive magnitude of *ϕ*_2_. With the FICO model, a list of features and weights will also give us these counterfactuals, but with LIME and SHAP, this is not the case.

It is initially surprising to say that FI explanations do not satisfy the goals of explainability, since they intuitively provide a sense of “how the model works”, and may make the operations of a model more transparent, understandable, and interpretable. However, while this kind of explanation may be valuable to designers, it is less valuable to model patients. Under the Evidence of Fariness view, this is because understanding the relative importance of actual features to each other does not tell us about what alternative features would have resulted in a better outcome. Rather than caring about the actual features which led a model to its decisions, model patients can and should care about alternative features which the model did not actually use to arrive at its decisions. Giving a FI explanation of a model like FICO will generate both explanations, but the FI explanations generated by LIME and SHAP will only provide the former.

Not only are FI explanations insufficient to provide evidence of fairness, they may also be unnecessary. Authors like Wachter et al. (2018) and Chou et al. (2021) have emphasized the way in which CS explanations do not rely on FI explanations:

In the existing [xAI] literature, “explanation” typically refers to an attempt to convey the internal state or logic of an algorithm that leads to a decision. In contrast, counterfactuals describe a dependency on the external facts that led to that decision …[Counterfactual explanations] are crafted in such a way as to provide a minimal amount of information capable of altering a decision, and they do not require the patient to understand any of the internal logic of a model in order to make use of it (Wachter et al., 2018).

While most xAI approaches tend to focus on answering why a certain outcome was predicted by a black-box, counterfactuals attempt to answer this question in another way by helping the user understand what features does the user need to change to achieve a certain outcome (Chou et al., 2021).

Both of these quotes suggest that it is entirely possible to provide good counterfactual statements about a model which is not “transparent” to patients. Indeed, there is some initial evidence that providing more detail about the process by which a model arrived at its decision might have a *negative* impact on model patients' satisfaction with an explanation (Lu et al., 2021).

### 5.2. Against AR explanations

It is obviously beneficial for people to be provided with recommendations for making improvements in their behavior. But some authors, like Ustun et al. (2019) and Karimi et al. (2021), have gone further and argued that actionable recourse is a primary normative and empirical motivation driving the value of explainability, and that CS explanations are insufficiently powerful on their own to provide the right kind of prescriptions for future behavior. This is because CS explanations (by definition) provide merely a set of states in the past which would have been judged by the model as acceptable, rather than a *procedure* that tells the model patient how to get to those states. In the words of Karimi et al.:

Counterfactual explanations…do not seem to fulfill one of the primary objectives of “explanations as a means to help a model patient *act* rather than merely *understand*.” [Counterfactual xAI methods] implicitly assume that the set of actions resulting in the desired output would directly follow from the counterfactual explanation. This arises from the assumption that “what would have had to be in the past” (retrodiction) not only translates to “what should be in the future” (prediction) but also to “what should be done in the future” (recommendation). We challenge this assumption and attribute the shortcoming of existing approaches to their lack of consideration for real-world properties, specifically the causal relationships governing the world in which actions will be performed.

Similarly, in the xAI method which McGrath et al. (2018) apply to credit scoring models, they include a weight to the distance metric between actual and counterfactual features with the goal of “obtaining counterfactuals that suggest a smaller number of changes or focus on values that are relevant to the individual and have historically been shown to vary”. This may actually be an excellent way to satisfy the focus on features which were under voluntary control in CS-explanations. However, McGrath et al. explicitly state that they are attempting to isolate features which are “historical and fixed”, like the number of delinquencies in the last six months, from features which can be changed in the future, like the amount one has in savings.

Regardless of the technical merits of the xAI methods presented by advocates of AR-explanations, we need to first consider the claim that recourse is an essential part of good explanations. The Evidence of Fairness view defended in this paper implies that this is not the case.^3^ Indeed, features like the number of delinquencies in the last six months are perfect examples of CS-explanations which provide positive evidence of fairness, although McGrath et al. are correct that they do not provide actionable recourse (unless we consider actionable recourse to be a recommendation like “have fewer delinquencies in the future”, but the authors don't seem to consider this to be a proper AR-explanation).

Here is where the empirical and the normative claims may importantly come apart. We strongly suspect that the primary psychological motivation for valuing explainable AI models is the evaluation of a past decision with respect to fairness, rather than the evaluation of future decisions with respect to benefit. However, the current empirical evidence may be insufficient to provide a compelling case for either view (Keane et al., 2021). In the absence of compelling empirical reasons, we will now consider a normative argument for why actionable recourse is not an ethical obligation for companies and governments to provide to model patients.

The normative argument draws on an old distinction between non-interference and benefit, where the former is morally obligatory and the latter is supererogatory (good and kind, but not morally obligated). Model patients have a moral right not to be unjustly denied or blocked from opportunities. The action of denying an applicant's request for credit (or providing a low credit score which has the same effect) is taking an action which denies opportunities to the applicant. It's true that not all denials of goods constitute an interference, and we can appeal to Rawls' (1971) distinction between primary and secondary goods, where primary goods are the kinds of resources which are necessary for any person to pursue their version of the good life (e.g., housing, employment, food and water, minimal safety, etc.), while secondary goods are the kinds of things which some people pursue and others do not. While denying someone access to a luxury good (through targeted marketing) may not genuinely be a blocking of opportunity, denying someone access to credit is plausibly a blocking of opportunity in the sense of preventing access to primary goods. Because a credit company has denied the applicant access to opportunities which include primary goods, the company has a burden of proof to demonstrate that this denial was justified, which is the grounds for the Evidence of Fairness view.

On the other hand, *assuming the past decision of the credit company was fair*, they have no moral obligations to provide applicants with the benefits of recommendations for improving their future outcomes. Once the company has demonstrated that they have not interfered with the applicant's rights, there are no additional obligations which could potentially compel them to provide benefits to that applicant *as an individual* (there may be general obligations to the public as a whole). These very broad obligations may exist in the form of public awareness campaigns, which aim at giving people a better understanding of what they need to do to improve their financial outcomes. But if we assume that the company *has not wronged* the applicant, then saying they owe her a personalized AR explanation is equivalent to saying that they owe every other person who they have not wronged a personalized AR explanation. There are no good normative reasons to justify this.

Nobody denies that model patients have an interest in beneficial recommendations, but there is a long list of items which people have an interest in but not a right to. For example, all job applicants have an interest in finding out the salaries of other employees, but this does not entail a right to this information. When companies have a competing interest against providing that information, they are entirely justified in not disclosing it. Similarly, there may often be a genuine cost for a company to implement xAI methods which generate AR explanations over and above CS explanations. In these cases, the company is justified in not using those xAI methods and still respecting the model patient's right to explainable decision-making.

## 6. Conclusions: Implications for regulation and technical metrics

This paper has defended the Evidence of Fairness view as a psychological and normative account of how people can and should evaluate satisfactory xAI methods. According to this view, xAI methods should be capable of generating CS explanations which provide positive evidence about controllable alternative states which would have led to a favorable outcome, and negative evidence about irrelevant group and behavioral alternative states which would not have led to a favorable outcome. We have proposed this as a psychological explanation about why people care about explainability, but have also provided normative reasons for why evidence of fairness is an ethical obligation. In this concluding section, we will consider some implications of this view for both regulations and technical metrics.

Recently there have been several efforts to pass legislation in the EU and US which would impose regulations on automated systems, including regulations about explainability. These include the AI Act in the US, and the Algorithmic Accountability Act in the EU. In addition, there are several existing laws in these regions which impose legal requirements for explainability, especially with respect to credit and lending. In the EU, the most relevant regulation is Article 22 of the General Data Protection Regulation (GDPR), which requires that firms provide “meaningful information about the logic involved, as well as the significance and the envisaged consequences of such processing for the data subject [model patient]”. In the US, the relevant regulation is Reg.B of the Equal Credit Opportunity Act (ECOA), which has required that patients receive “specific reasons” for a decision that caused some adverse impact.

There has been intense debate regarding what “meaningful information” and “specific reasons” mean in the context of automated systems (Wachter et al., 2018; Casey et al., 2019). Wachter et al. (2018) note that the GDPR is ambiguous about what types of information counts as meaningful, and could be interpreted as what we've identified as FI, CS, or AR explanations. They note that the statements about explainability are largely ambiguous between what we're calling FI and CS explanations, but that AR explanations are not explicitly included: “Using explanations as a guide to altering behavior to receive a desired automated decision is not directly addressed in the GDPR” (Wachter et al., 2018). If the Evidence of Fairness view is correct, it could be used to not only interpret what type of explainability is relevant for satisfying the values of model patients, but also clarifying standards for explainability in future regulations.

For engineering teams who are designing AI models and their associated xAI methods, the Evidence of Fairness view can help to provide an evaluation procedure for outputs of these methods. Namely, methods like LIME and SHAP may be interesting for the purpose of understanding a model, but for the purpose of justifying it, they are insufficient because they fail to generate the right kinds of CS explanations. Similarly, actionable recourse xAI methods (Joshi et al., 2019; Ustun et al., 2019; Karimi et al., 2021) may be interesting for the purpose of public welfare, but they are not necessarily related to justifying past decisions of a model, and can potentially detract from other legal and ethical obligations which an organization has to its stakeholders.

A more complicated issue relates to the common use of group fairness metrics such as demographic parity to evaluate the fairness of an AI model. As noted in Section 3, there is often a conflict between demanding that a model satisfy both group parity and evidence of procedural fairness. One way to resolve this conflict is to value one type of fairness over the other, and explain to stakeholders why this is being done. Another resolution is to make adjustments to the type of group parity metrics which are used, only using those which are consistent with procedural fairness, such as group parity conditionalized on qualification, which Hardt et al. (2016) call “equalized odds” or “equality of opportunity”. A third resolution is to make adjustments to the type of CS-explanations which are used, where an organization might provide CS explanations as evidence that no discrimination has occurred against historically disadvantaged groups, but refuse to provide CS-explanations as evidence that no discrimination has occurred against historically privileged groups (so-called “reverse discrimination”). Deciding which way to resolve this *inter-value* conflict is beyond our scope, but an important problem for AI ethics.

Finally, in addition to the genuine conflict between explainability/procedural fairness and group parity, there is also a conflict between explainability and corporate privacy. Companies have rights to keep some amount of detail about their models private, and providing too much information about the models may violate those rights. Companies also have a legitimate concern with model patients taking advantage of explainability to take measures that artificially inflate their credit score. This is another example of inter-value conflicts which must be resolved by industry standards and (if needed) regulations.

The subfield of xAI is still just beginning. As Keane et al. (2021) correctly observe, there is currently a lack of connection between the design of counterfactual xAI methods and the attitudes and values of model patients, but there is movement in this direction. We encourage researchers to investigate the predictions of the Evidence of Fairness view and its alternatives, regarding what kinds of counterfactual changes are viewed as most relevant to assessments of a fair decision, and how to implement these counterfactual changes into an xAI method. We also encourage those who design xAI methods to work in the direction of both social science and normative theories, and aim toward a reflective equilibrium between these two in identifying what outputs of an xAI method constitute an acceptable and permissible explanation.

## Author contributions

The author confirms being the sole contributor of this work and has approved it for publication.

## Footnotes

^1^For example, Salmon (1994) advocates a “mechanistic” account of causal explanation (corresponding to FI), where some claim (call this *E*) explains a set of observations (call these *O*) to the extent that *E* provides an account of the mechanisms which brought about *O*. Lewis (1973) argues for a counterfactual account, where *E* explains *O* to the extent that *E* describes what would have been true in worlds where *O* was and was not observed. Finally, Woodward (2003) argues that *E* explains *O* to the degree that it allows us to manipulate *O*, that is, to determine by choice when *O* will or will not occur in the future.

^2^Formally, this is just the solution to: *max*[*u*[*e*(*A*), *i*(*A*)]], where *A* are the set of all possible future actions, *e*(*A*) is the ease of accomplishing those actions, *i*(*A*) is the positive impact of those actions on the FICO score, and *u*(*x*) is some welfare aggregation function.

^3^It is worth nothing that even some advocates of AR explanations, like Joshi et al. (2019), acknowledge that their xAI methods are not intended to satisfy legal or ethical obligations to model patients.
